# MGL2^+^ Dermal Dendritic Cells Are Sufficient to Initiate Contact Hypersensitivity *In Vivo*

**DOI:** 10.1371/journal.pone.0005619

**Published:** 2009-05-19

**Authors:** Yosuke Kumamoto, Kaori Denda-Nagai, Satoshi Aida, Nobuaki Higashi, Tatsuro Irimura

**Affiliations:** Laboratory of Cancer Biology and Molecular Immunology, Graduate School of Pharmaceutical Sciences, the University of Tokyo, Tokyo, Japan; New York University School of Medicine, United States of America

## Abstract

**Background:**

Dendritic cells (DCs) are the most potent antigen-presenting cells in the mammalian immune system. In the skin, epidermal Langerhans cells (LCs) and dermal dendritic cells (DDCs) survey for invasive pathogens and present antigens to T cells after migration to the cutaneous lymph nodes (LNs). So far, functional and phenotypic differences between these two DC subsets remain unclear due to lack of markers to identify DDCs.

**Methodology/Principal Findings:**

In the present report, we demonstrated that macrophage galactose-type C-type lectin (MGL) 2 was exclusively expressed in the DDC subset in the skin-to-LN immune system. In the skin, MGL2 was expressed on the majority (about 88%) of MHCII^+^CD11c^+^ cells in the dermis. In the cutaneous LN, MGL2 expression was restricted to B220^−^CD8α^lo^CD11b^+^CD11c^+^MHCII^hi^ tissue-derived DC. MGL2^+^DDC migrated from the dermis into the draining LNs within 24 h after skin sensitization with FITC. Distinct from LCs, MGL2^+^DDCs localized near the high endothelial venules in the outer T cell cortex. In FITC-induced contact hypersensitivity (CHS), adoptive transfer of FITC^+^MGL2^+^DDCs, but not FITC^+^MGL2^−^DCs into naive mice resulted in the induction of FITC-specific ear swelling, indicating that DDCs played a key role in initiation of immune responses in the skin.

**Conclusions/Significance:**

These results demonstrated the availability of MGL2 as a novel marker for DDCs and suggested the contribution of MGL2^+^ DDCs for initiating CHS.

## Introduction

Dendritic cells (DCs) are highly potent antigen presenting cells (APCs) capable of initiating immune responses. In the skin, Langerhans cells (LCs) in the epidermis form a network for surveillance for antigens. LCs are known to express Langerin, a C-type lectin which contributes to formation of Birbeck granules [1]–[3]. Besides the LCs, normal skin contains another type of DCs in the dermis [4]. In the initiation phase of immune responses in the skin, both the LCs and dermal DCs (DDCs) are thought to migrate into the skin-draining lymph nodes (LNs) to present antigens to T cells [5], [6]. However, functional and phenotypic differences between LCs and DDCs are still controversial.

In spite of their clear definition of DDCs based on their localization in the skin, there is no straightforward way to detect DDCs in murine LNs due to the lack of DDC-specific marker, making it complicated to correctly understand their roles in immune responses in comparison to LCs. In LNs, at least five different subsets of DCs have been identified in mice based on their surface markers, two of which are restricted to the skin-draining and mesenteric LNs, and are therefore thought to correspond to LCs or DDCs in the skin [7]–[14]. In these previous studies, however, difference between LCs and DDCs in the skin-draining LNs was ambiguous because the distinction was based on the differential intensity of markers such as CD8α, CD11b, and CD205 [10].

Contact hypersensitivity (CHS) is one of the well-characterized immune responses in the skin. Although LCs have long been believed to be responsible for CHS initiation as APCs [15], [16], recent studies on *in vivo* LC-depletion in mice reported a significant CHS response, indicating dispensability of LCs and contribution of DDCs to the CHS induction [12], [17], [18]. However, the interpretation of their results should be much more complicated after the recent discovery of Langerin^+^DDCs [19]–[21]. Thus, a role for DDCs in the induction of immune responses was only indirectly suggested by depleting LCs or by painting of a fluorescent dye in bone marrow-chimeric mice [22]. The lack of a direct proof is at least in part due to the absence of specific markers for DDCs.

Macrophage (MØ) galactose-type C-type lectins (MGLs) are a family of type II C-type lectins expressed in connective tissue MØs and in bone marrow-derived DCs [23], [24]. The number of MGL^+^cells, originally thought to be MØs, in the draining LNs was previously shown to correlate to the severity of CHS following epicutaneous application of a hapten, fluorescein isothiocyanate (FITC) [25]. We recently found that there are two MGL genes in mice and cloned a novel murine *Mgl2*, that made previously known *Mgl* as *Mgl1* [26]. Although analysis of mRNA expressions in mouse tissues and cell lines suggested that the *Mgl2* gene was expressed in a cell population similar to that expressing MGL1 [26], a detailed characterization of cells expressing MGL2 was not clear, since MGL-specific monoclonal antibodies (mAbs) used previously turned out to recognize a common epitope for MGL1 and MGL2 (mAb LOM-14 and mAb ER-MP23) except for mAb LOM-8.7 specific for MGL1 [26]–[28].

Development of mAb specific for MGL2, URA-1, enabled us to characterize the cell populations expressing MGL2 in the skin and the cutaneous LNs. MGL2 was found to be highly restricted to DDCs in the skin and the LNs. We observed rapid accumulation of MGL2^+^DDCs, which localized in the outer T cell cortex closely associated with the high endothelial venule (HEV)-related reticular structure, in the draining LNs within 24 h of sensitization with FITC. These cells were sufficient to induce a CHS response when adoptively transferred to naïve mice. In contrast, adoptive transfer of FITC^+^MGL2^−^DCs isolated from the draining LNs 4 days after sensitization did not induce CHS. These results provide a concrete evidence that hapten-incorporated DDCs are sufficient to induce CHS *in vivo*.

## Materials and Methods

### Mice and FITC sensitization

Five- to seven-week-old specific pathogen-free Balb/c mice of both sexes were purchased from Charles River Japan (Yokohama, Japan) or SLC Japan (Hamamatsu, Japan). They were fed and housed according to the guidelines of the Ministry of Education, Culture, Sports, Science and Technology of Japan. The protocols of the animal experiments were approved by the Animal Experiment Committee of the Graduate School of Pharmaceutical Sciences of the University of Tokyo guided by the Bioscience committee of the University of Tokyo. In the CHS experiments, mice were painted with 0.5% FITC (w/v) in a 1∶1 mixture of acetone and dibutylphthalate (ADBP) on the shaved brachial (100 µl/site) and/or abdominal (100 µl/site) skin.

### Histochemistry

All mAbs used in the present study were purchased from eBioscience (San Diego, CA) unless otherwise indicated. mAbs to MGL1 (clone LOM-8.7) and MGL2 (clone URA-1) were purified and labeled as previously described [29], [30]. The details in the mAb URA-1 will be described elsewhere (Denda-Nagai et al, manuscript in preparation). In fluorescent microscopy, frozen sections were incubated with mAbs LOM-8.7, URA-1, LOM-14, MECA-79 (BD Biosciences, Franklin Lakes, NJ), ER-TR7 (Serotec, Oxford, UK) or anti-Langerin (eBioRMUL.2) followed by treatment with Cy5-conjugated mouse anti-rat IgG (H+L) (Jackson ImmunoResearch, West Grove, PA). Propidium iodide was used for the counter-staining. For double staining experiments, samples were sequentially treated with biotinylated mAbs LOM-8.7, URA-1, anti-CD11b (M1/70), or anti-F4/80 (BM8). The biotinylated mAbs were visualized by Alexa Fluor 568-conjugated streptavidin (Invitrogen, Carlsbad, CA) and were observed with a confocal microscope (MRC-1024, Bio-Rad, Hercules, CA). In some experiments, biotinylated mAbs LOM-14, anti-CD11c (N418), anti-B220 (RA3-6B2) and anti-MHCII (M5/114.15.2) were used in combination with alkaline phosphatase-conjugated streptavidin (Vector Laboratories, Burlingame, CA) developed by Histomark Red (Kirkegaard & Perry, Geithersburg, MD) and observed under a light microscope (TMD-300, Nikon).

### Flow cytometry

LNs were teased and digested in RPMI1640 medium supplemented with 10% fetal calf serum, 2 mg/ml collagenase (Wako Pure Chemical, Osaka, Japan) and 50 µg/ml DNaseI (Roche, Basel, Switzerland) for 30 min at 37°C. Skin pelt after removing the adipose was incubated with 0.8% trypsin (BD Biosciences) in PBS. The epidermis and the dermis were then separately digested with 6 mg/ml collagenase and, in the case of the dermis, with additional 2 mg/ml hyaluronidase (MP Biomedicals, Irvine, CA). To stain intracellular molecules, cells were permeabilized with FIX&PERM (An Der Grub Bioresearch, Kaumberg, Austria). In some cases, CD11c^+^cells were isolated by magnetic cell sorting (MACS) (Miltenyi Biotec, Gladbach, Germany) according to the manufacturer's protocol. The ratio of CD11c^+^cells in the purified fraction was over 95%, which was confirmed by phycoerythrin (PE)-labeled anti-CD11c mAb in each experiment. The cells were incubated with mixtures of biotinylated mAb LOM-8.7 or mAb URA-1 and PE-labeled mAbs against CD4 (GK1.5), CD8α (53-6.7), CD11b, CD11c, CD86 (GL1), B220, F4/80, Ly6G (RB6-8C5), MHCII or CD40 (1C10, BioLegend, San Diego, CA) followed by treatment with streptavidin-conjugated allophycocyanin (eBioscience). In some experiments, FITC-conjugated anti-CD11c mAb, Alexa Fluor 488-conjugated anti-Langerin, PE-conjugated anti-CD205 (NLDC-145, Miltenyi) and allophycocyanin-labeled mAb URA-1 were used. Dead cells were stained and gated out with 7-aminoactinomycin D (eBioscience). The cells were analyzed by FACSAria cell sorter with FACSDiVa software (BD Biosciences).

### In vivo migration of DC

Mice were shaved in the side-abdominal skin (approximately 2×2 cm^2^) and painted with 50 µl of ADBP. The cell suspensions from the site of skin irritation and the draining inguinal LNs were analyzed by flow cytometry. In the pertussis toxin (PTX) treatment, mice were injected with 0.5 µg of PTX (ListBiological Laboratories, Campbell, CA) into the front footpad, then 30 µl of FITC/ADBP solution was painted on the tip of the same footpad. The draining brachial and axillary LNs 24 h after sensitization were excised to prepare the cell suspensions.

### Adoptive transfer and elicitation of CHS

CD11c^+^cells were isolated by MACS from the draining LN 24 h or 4days after sensitization, then FITC^+^ MGL2^+^cells or FITC^+^ MGL2^−^cells were sorted by FACSAria cell sorter. The sorted cells (7.5×10^4^ or 5×10^4^ cells/recipient) were resuspended in 30 µl each of Hanks' balanced salt solutions and injected into the footpad of naive mice. The same number of cells fixed with 2.5% (v/v) glutaraldehyde was used as a control. Six days later, the recipient mice were painted with 20 µl of 0.5% FITC/ADBP on the dorsal right ear skin. The left ear was treated with the vehicle alone as a control. As negative and positive controls for sensitization, naive mice and mice painted with 200 µl of 0.5% FITC/ADBP 6 days prior to sensitization were used. The FITC-specific ear swelling was defined as follows: (right ear thickness at 24 h after elicitation - right ear thickness before elicitation)-(left ear thickness at 24 h after elicitation-left ear thickness before elicitation).

### Statistic Analysis

All experiments were independently performed at least three times, except the glutaraldehyde fixation and FITC^+^MGL2^−^DC controls in the adoptive transfer experiments, which were independently performed twice. Data were compared by two-sided Student's *t* test and presented as mean±standard deviation (SD).

## Results

### Expression of MGL1and 2 in a major DDC subset in the skin was shown by antibody bindings

**Figure 1:**
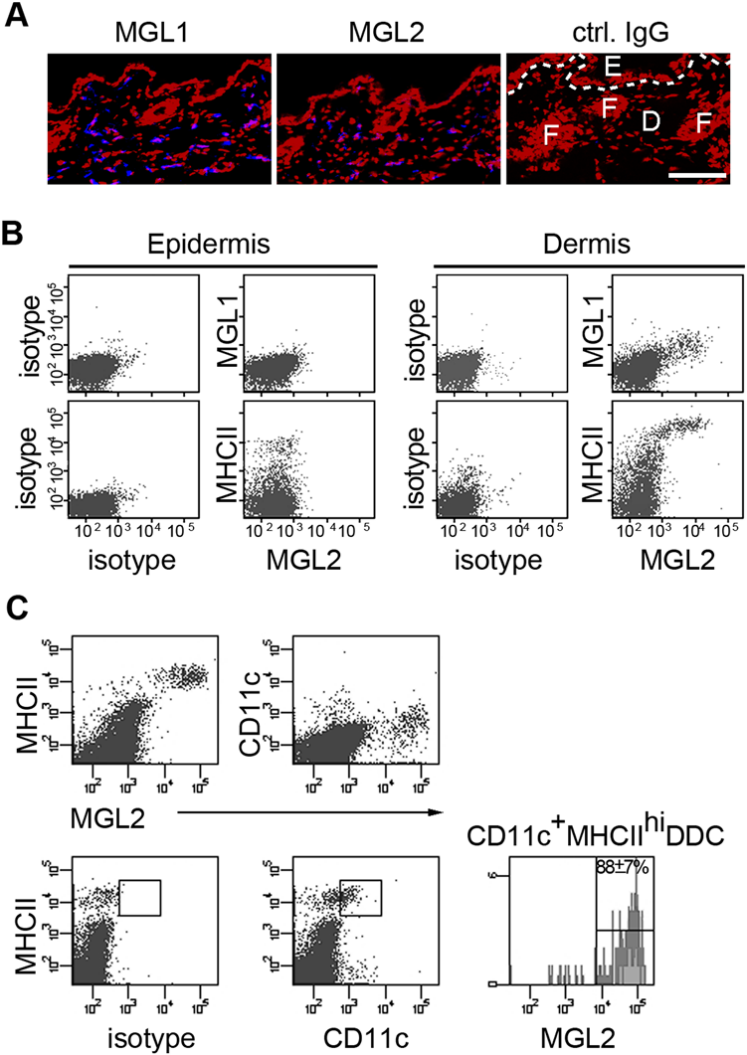
Expression of MGL1 and MGL2 in a DDC subset in skins of naive mice. (A) Binding of anti-MGL1 and anti-MGL2 mAbs to cryosections of skin (blue in each panel). The dashed line in the control panel (Ctrl. IgG) indicates the dermal-epidermal junction. Nuclei are shown in red. Scale bar, 100 µm. E: epidermis, D: dermis, F: hair follicle. (B) Surface staining by anti-MGL1 and anti-MGL2 mAbs of epidermal and dermal cell suspensions. (C) Intracellular staining of CD11c and surface staining of MHCII and MGL2 in the dermal cell suspension. Virtually all MGL2^+^ cells expressed CD11c. The proportion of MGL2^+^ cells in MHCII^hi^ CD11c^+^ DDC (cells in the rectangular gate) was 88.5±6.8%.

Bindings and localization of mAb LOM-8.7(anti-MGL1) and mAb URA-1(anti-MGL2) in skin of naïve mice were immunohistochemically examined. Binding sites of these mAbs showed similar distribution patterns within the dermis, though more MGL1^+^cells appeared to be present than MGL2^+^cells (Figure 1). These mAbs did not bind any cells in the epidermis, indicating the absence of MGL1 or 2 in epidermal LCs (Figure 1A) as shown in our previous work with a MGL1/2 cross-reactive mAb (23). By flow cytometric analysis of the dermal and epidermal cell suspensions, cells bound by mAb LOM-8.7 or mAb URA-1 were observed only in cell suspensions from the dermis (Figure 1B). Binding of mAb URA-1 was observed with the cells, to which mAb LOM-8.7 and mAb specific for MHCII strongly bound (Figure 1B). Higher levels of MHCII were found on the cells reactive to mAb URA-1 compared to the LCs from the epidermis, suggesting that MGL2^+^ cells potentially function as APCs (Figure 1B). Intracellular CD11c was also determined after permeabilization, because the cell surface CD11c is known to be cleaved by trypsinization [31]. The expression of MGL2 was shown to be restricted to ∼88% of CD11c^+^MHCII^hi^ DDCs (Figure 1C). The MGL2^−^CD11c^+^MHCII^hi^ cells (12±7% in dermal CD11c^+^MHCII^+^cells) may represent migrating LCs, undifferentiated DDC precursors, or another DDC subset [1], [19]–[21], [32].

### MGL2^+^DCs migrate to the cutaneous LNs after FITC sensitization

**Figure 2:**
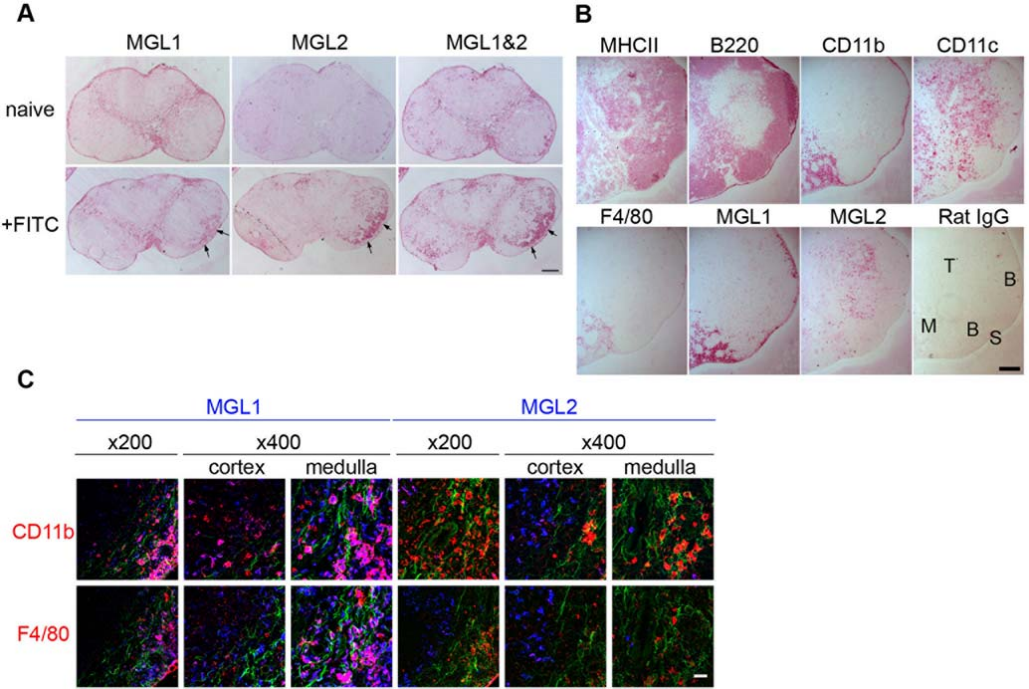
Distribution of MGL2^+^ cells in draining LNs during the sensitization with FITC revealed by immunohistochemical analysis. (A) Serial sections of LNs stained with anti-MGL1, anti-MGL2 or anti-MGL1/2 cross-reactiv mAbs. MGL1 was strongly stained in the sinuses in both naive and FITC sensitized (24 h after sensitization) LNs. Arrows indicate the increased signals in the cortex after the sensitization, though the anti-MGL1 staining in the cortex was weaker than in the sinuses. Scale bar, 250 µm. (B) Serial LN sections 4 h after sensitization stained with mAbs to MØ/DC markers. Restricted localization of MGL2^+^ cells to the outer T cell cortex was unique compared to other APCs (MHCII) including B cells (B220), MØs (CD11b and F4/80), DCs (CD11c) or MGL1^+^ cells. S: subcapsular sinus, M: medulla, T: T cell cortex, B: B cell follicle. Scale bar, 200 µm. (C) Confocal microscopic observations of the distributions of MGL1 (mAb LOM-8,7) or MGL2 (mAb URA-1) (blue in each panel) with MØ markers (red) in the LN 24 h after FITC sensitization. FITC used is shown in green. Scale bar, 25 µm (×400).

We subsequently examined bindings of mAbs specific for MGL1 or MGL2 to the skin-draining LNs. Distribution of binding sites for mAb LOM-8.7 was mainly observed in the medullary and subcapsular sinuses, which was a similar distribution seen by MGL1/2-cross reactive mAb LOM-14 previously (Figure 2A). Distribution of binding sites of mAb URA-1 was restricted to the cortical areas (Figure 2A). As previously shown by the use of mAb LOM-14 [25], a larger number of cells reactive with mAb URA-1 were observed in the T cell cortex after FITC sensitization (Figure 2A). Binding of mAb LOM-8.7 specific for MGL1 was found both in the sinuses and in the cortex. The distributions of cells reactive with these mAbs were compared with distributions of cells reactive with mAbs to other MØ/DC markers in the draining LNs 4 h after FITC sensitization. Immunohistochemical staining with mAb LOM-8.7 indicated that the level of MGL1 expression of the cells in the cortical area was lower than that of the cells in the sinusoidal area (Fig, 2A and B). The MGL1 distribution in the sinusoidal areas overlapped with that of CD11b and F4/80 and these cells probably represent sinusoidal MØs (Figure 2B) consistent with our previous reports [23], [33]. Colocalization of CD11b or F4/80 with MGL1 in relatively large cells in the medullary sinus was further confirmed by confocal microscopy (Figure 2C). In the cortex, distribution of cells reactive with mAb LOM-8.7 and those with mAb URA-1 was similar to that of cells bound by anti-MHCII and anti-CD11c mAbs, indicating that MGL1^+^/MGL2^+^ cells represent DCs distributing within the T cell areas (Figure 2B). While cells bound by anti-MHCII and anti-CD11c mAbs scattered throughout the cortex, binding of mAb URA-1 was highly restricted to the cells in outer region within the cortex, suggesting that MGL2^+^ cells represented a distinct population in DC subsets. Unlike the binding sites of mAb LOM-8.7, the binding sites of mAb URA-1 did not overlap with CD11b or F4/80 in the medullary sinus. In contrast, overlap of mAb URA-1 binding with these markers was observed in the cortex where the majority of MGL2^+^ cells localized (Figure 2C). These results indicate that MGL2 is expressed in a relatively homogenous population whereas MGL1 associates with several populations including the MGL2^+^ cells in the skin-draining LN.

### Flow cytometric analysis indicated that mAb URA-1 binding was limited to CD8α^lo^CD11b^+^CD205^int^ DDCs in the subcutaneous LNs

**Figure 3:**
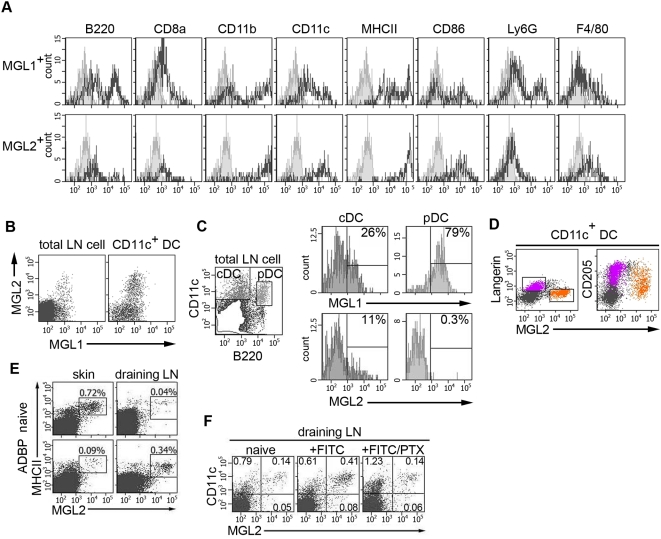
Surface phenotype of MGL1^+^ and MGL2^+^ cells in naive peripheral LNs revealed by the flow cytometric analysis. (A) Surface marker staining on cells gated on MGL1^+^ or MGL2^+^ populations in naive cutaneous LN. The gray peak in each panel indicates binding of rat IgG2a as a negative control. (B) Double staining of anit-MGL1 and anti-MGL2 mAbs on the total LN cells (left) or MACS-purified CD11c^+^ DCs (right). (C) Surface staining by anti-MGL1 and anti-MGL2 mAbs on B220^+^CD11c^int^ pDCs and B220^−^ CD11c^+^ cDCs. Each DC subset was gated as in the left panel. (D) Intracellular staining of Langerin and surface staining of CD205 and MGL2 in MACS-purified CD11c^+^ DCs. Langerin^+^ and MGL2^+^ cells were shown in purple and orange respectively as gated in the left panel. (E) Flow cytometric analysis of the ratio of MGL2^+^ DC (gated) in the naive and ADBP-treated (24 h after the irritation) skins and LNs. Note that MGL2 signal was bright in all samples, suggesting that MGL2 expression was stable upon migration. (F) Effect of PTX on the ratio of MGL2^+^ DDC after FITC sensitization. The increase in the number of MGL2^+^CD11c^+^ DC induced by FITC (+FITC) was significantly reduced (*p*<0.05) by concurrent administration of PTX with FITC painting (+FITC/PTX).

The surface phenotype of cells reactive with anti-MGL1 or anti-MGL2 mAbs in the skin-draining LNs was further examined by flow cytometry. As suggested by the results of immunohistochemical examinations, the mAb LOM-8.7-binding MGL1^+^ population was found to contain several subpopulations, while mAb URA-1-binding MGL2^+^ population represented a homogenous group of cells (Figure 3A). The MGL1^+^ population contained a B220^+^, CD11b^−^, CD11c^int^, MHCII^lo^, CD86^−^, Ly6G^+^ subpopulation, while the MGL2^+^ population did not (Figure 3A). Double labeling analysis showed that all MGL2^+^cells were reactive with mAb LOM-8.7 and that a distinct population was reactive with mAb LOM-8.7 but not with mAb URA-1 (Figure 3B).

The MGL1^+^MGL2^−^ population, which was B220^+^, CD11c^int^, Ly6G^+^ and MHCII^lo^, apparently corresponded to the previously published phenotype of plasmacytoid DCs (pDCs) [8], [34]. This was confirmed by the fact that pDCs (B220^+^CD11c^int^) only expressed MGL1 but not MGL2 (Figure 3C). Although we clearly observed MGL1^+^MGL2^−^MØs by immunohistochemistry (Figure 2C), we could not detect any F4/80^hi^ typical MØs by flow cytometry probably due to difficulties in isolating MØs from the LNs as single cell suspensions. The conventional DC (cDC) fraction in the MGL1^+^cell population showed a similar phenotype to the MGL2^+^ population, which was B220^−^, CD8α^lo^, CD11b^hi^, CD11c^+^, MHCII^hi^, CD86^+^, Ly6G^−^ and F4/80^+^ (Figure 3A). This surface phenotype indicated that the MGL1^+^MGL2^+^ population represented a subset of interstitial DC according to previous reports [7], [9], [10]. Therefore, the MGL1^+^ population was likely to contain interstitial DCs, pDCs and MØs and the MGL2^+^ population consisted of interstitial DCs, which also expressed MGL1. The surface phenotype of MGL2^+^DDCs in the skin (Figure 1) and in the LNs (Figures 2 and 3) was similar in C57BL/6 mice (data not shown).

Considering a previous report that interstitial DCs in the skin-draining LNs were derived from the skin [11], the expression of Langerin in MGL2^+^DCs was examined to judge whether they were derived from LCs or DDCs. The MGL2^+^ population was Langerin^−^CD205^int^ by the intracellular Langerin staining (Figure 3D), indicating that they represented a population derived from DDCs. Only a minor subset of CD11c^+^ DCs seemed to be positive with both MGL2 and Langerin, perhaps corresponded to the Langerin^+^DDCs [19]–[21] reported previously as a minor DC subset [28]. The number of MGL2^+^ DCs in the skin and in the corresponding draining LNs was compared before and after irritation of the skin with ADBP. MGL2^+^ DCs decreased in the skin and increased in the draining LNs concomitantly after irritation with ADBP (Figure 3E), suggesting that the MGL2^+^ population migrated from the dermis to the draining LN. In addition, the expression level of MGL2 apparently remained stable after migration (Figure 3E).

The migration of skin-resident DCs to the subcutaneous LNs was known to be chemokine- and its downstream G protein-dependent and therefore blocked by G protein inhibitor PTX. As shown in Figure 3E, increase in the number of MGL2+ cells was not observed in the draining LN when PTX was *s.c.* injected into the footpad prior to the administration of FITC/ADBP. The results strongly suggest that migration of MGL2^+^ DDCs is chemokine-dependent. Furthermore, these results indicate that MGL2 is useful as a marker to identify DDCs in the skin-to-LN immune system.

### MGL2^+^DDCs express high levels of costimulatory molecules even under nonstimulated conditions

**Figure 4:**
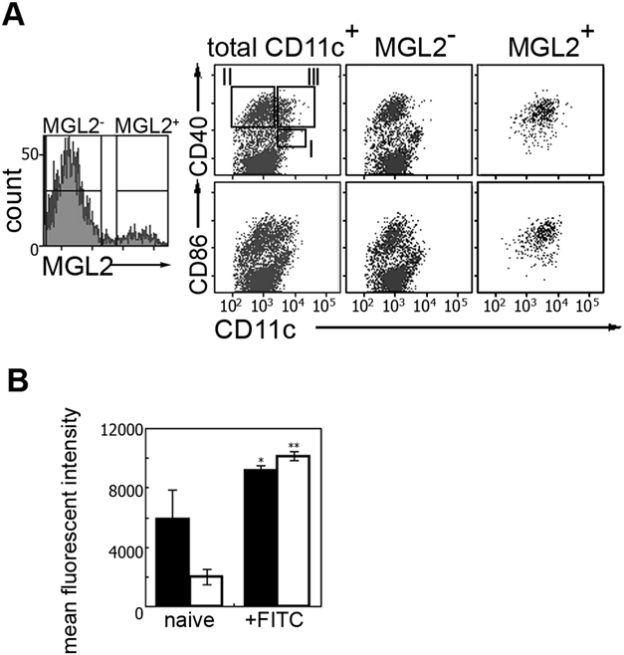
Expression of co-stimulatory molecules in MGL2^+^ DDC revealed by the flow cytometric analysis. (A) CD40 and CD86 staining on MGL2^+^ DDC in naive cutaneous LNs. MACS-purified CD11c^+^ DCs were sub-divided into MGL2^+^ DDCs and MGL2^−^ DCs (left panel). Populations I-III indicate known cDC subsets in murine LNs. Note that MGL2^+^ DDCs were exclusively assigned to population III (CD11c^hi^CD40^hi^ DC). (B) Mean fluorescent intensity of CD40 (filled bars) and CD86 (open bars) staining of MGL2^+^ DDCs after FITC sensitization in the LN. *p<0.05, **p<0.00005. Mean±SD obtained from three mice is shown.

To further characterize the MGL2^+^DDCs, we compared the surface phenotypes of these cells with MGL2^−^DCs in LNs. Naive CD11c^+^DCs isolated by MACS were separated into two groups based on their expression of MGL2. The expression levels of CD8α, CD11c, CD40, and CD86 were examined in these two populations. As previously described [9], the total CD11c^+^ DCs were further divided into three groups by the expression levels of CD11c and CD40 (population I: CD40^int^CD11c^hi^, population II: CD11c^int^CD40^hi^, and population III: CD11c^hi^CD40^hi^) (Figure 4A). MGL2^+^ DDCs were highly homogeneous and consisted of only CD11c^hi^CD8α^lo^CD40^hi^CD86^hi^ cells, which corresponded to population III in the previous report (Figure 4A) [9]. The high expression levels of CD40 and CD86 in MGL2^+^DDCs indicated that these cells were matured by definition at the steady state as demonstrated previously for migratory CD11c^+^MHCII^hi^ DCs (Figure 4A) [35]. It was previously suggested that CD11c^hi^CD40^hi^ DCs (population III) were derived from epidermal LCs because of the presence of cells containing Birbeck granules, though their frequency seemed to be small [9]. However, only a few MGL2^−^ cells belonged to population III, suggesting that most cells in population III were DDCs (Figure 4A). Interestingly, the expression of both CD40 and CD86 on MGL2^+^ DDCs was higher in the draining LNs 24 h after FITC sensitization than in the naïve LNs (Figure 4B). Because expression levels of MGL2 on DDCs were not affected by migration, and MGL2^+^DDCs in the non-sensitized LNs are likely to represent a “steady-state migratory” DC population (Figure 3E), these results indicated that newly-migrated “sensitization-induced migratory” DDCs express higher levels of co-stimulatory molecules than MGL2^+^DDCs that were already in the LNs by steady-state migration at the time of sensitization, as was suggested in the previous report [35]. These results suggest that MGL2^+^DDCs have a “semimature” phenotype even under steady state conditions and further upregulate their co-stimulatory molecule expression upon sensitization.

### MGL2^+^DDCs incorporate FITC and accumulate in the draining LNs within 24 h after FITC sensitization

Recent studies indicated that a migrant DC population loaded with haptens reached the draining LNs earlier than LCs after CHS sensitization [12], [22]. The immigration of such migrant DCs peaked between 24 to 48 h after sensitization, 2 or 3 days prior to the migration of LCs [12], [22]. Because of the absence of Langerin expression and the incorporation of skin-painted haptens, it was speculated that these migrant DCs were derived from the dermis. However, resident DCs in LNs might also take up soluble antigens drained directly to the LNs [11]. In addition, inflammatory monocyte-derived DCs recruited to the skin or to the skin-draining LNs from the blood upon skin inflammation were also potential APCs in the skin-draining LNs [36]–[38]. Therefore, the presence of hapten alone might not indicate that the cells were skin-derived.

**Figure 5:**
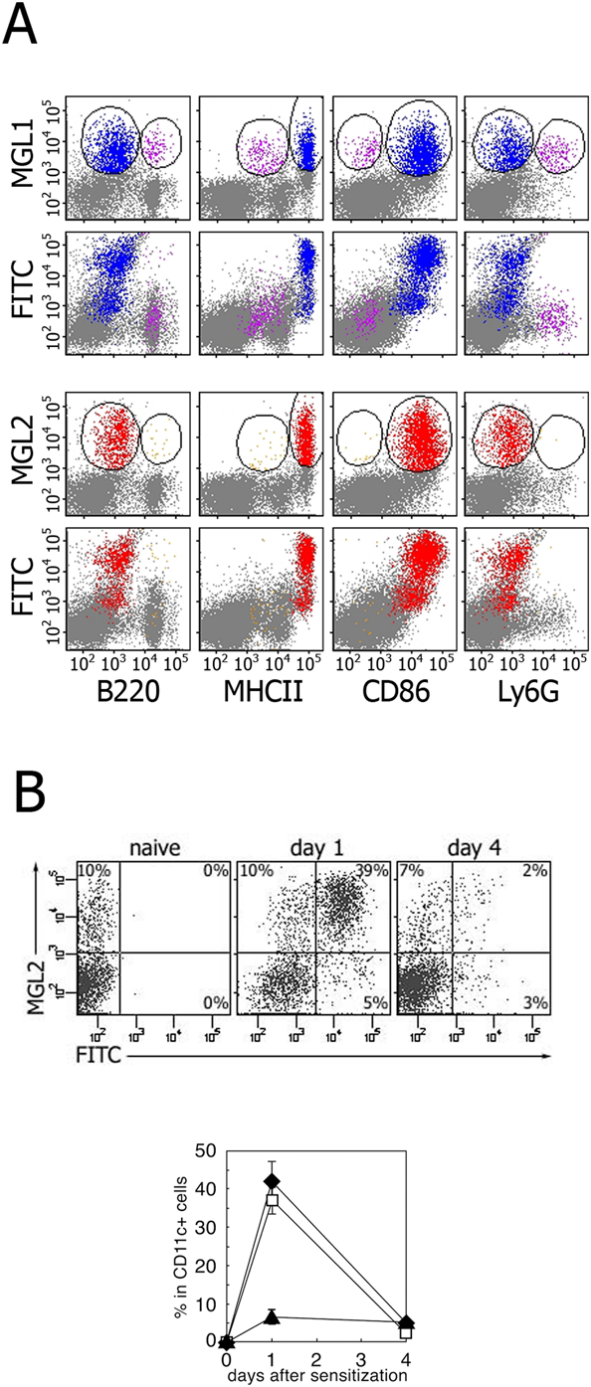
Hapten incorporation and migration kinetics of MGL2^+^ DDC after FITC sensitization revealed by the flow cytometric analysis. (A) FITC fluorescence associated with MGL2^+^ DDCs in LNs. MGL1^+^ cDC (blue) or MGL2^+^ DDCs (red), but not MGL1^+^ pDCs (purple) or cells nonspecifically bound by mAb URA-1 (orange) incorporate FITC. (B) Kinetics of MGL2^+^ DDCs in LNs after FITC sensitization. Top panels indicate the FITC fluorescence and staining with anti-MGL2 mAb on DCs purified from naive LNs (left) or LNs 24 h (middle) or 96 h (right) after sensitization. The bottom panel shows the ratio of total FITC^+^ DCs (closed diamond), FITC^+^ MGL2^+^ DDCs (open square) and FITC^+^ MGL2^−^ DCs (closed triangle) calculated by each quadrant shown in top panels. The data are shown as Mean±SD.

To answer this question, we attempted to examine the antigen uptake and time course of arrival of these cells in LNs using MGL2 as a marker for the DDC subset. First, incorporation of FITC into MGL1^+^ cells and MGL2^+^ DDCs, both of which accumulate in the LNs 24 h after sensitization, was examined [25]. At 24 h after sensitization, MGL2^+^ DDCs as well as MGL1^+^ cDCs, which include the MGL2^+^ DDC population (Figure 3B), were found to contain FITC, while MGL1^+^ pDCs did not (Figure 5A). The proportion of MGL2^+^ DDCs was about 10% among CD11c^+^ DCs in the naive LNs, and increased up to 50% on day 1, then decreased to the steady state level by day 4 (Figure 5B). The percentage of the FITC^+^ MGL2^+^ DDCs was the highest on day 1, as was also the case with the total FITC^+^ DC percentage in the LNs, while the ratio of FITC^+^ MGL2^−^ DCs on day 1 was similar to that on day 4, indicating that MGL2^+^ DDCs migrated rapidly compared to the MGL2^−^ DCs (Figure 5B), consistent with the non-LC migrants in previous reports [12], [22]. The presence of FITC (Figure 5) and the absence of Ly6G expression (Figure 3A) in MGL2^+^ DCs clarifies that MGL2^+^ DCs are a distinct population from inflammatory monocyte-derived DCs [37], [39], [40]. These results suggest that MGL2^+^ DDCs with antigens arrived earlier at the draining LNs and were more likely to initiate immune responses than LCs in CHS sensitization.

### MGL2^+^DDCs localize near the high endothelial venules in naive and sensitized LNs

**Figure 6:**
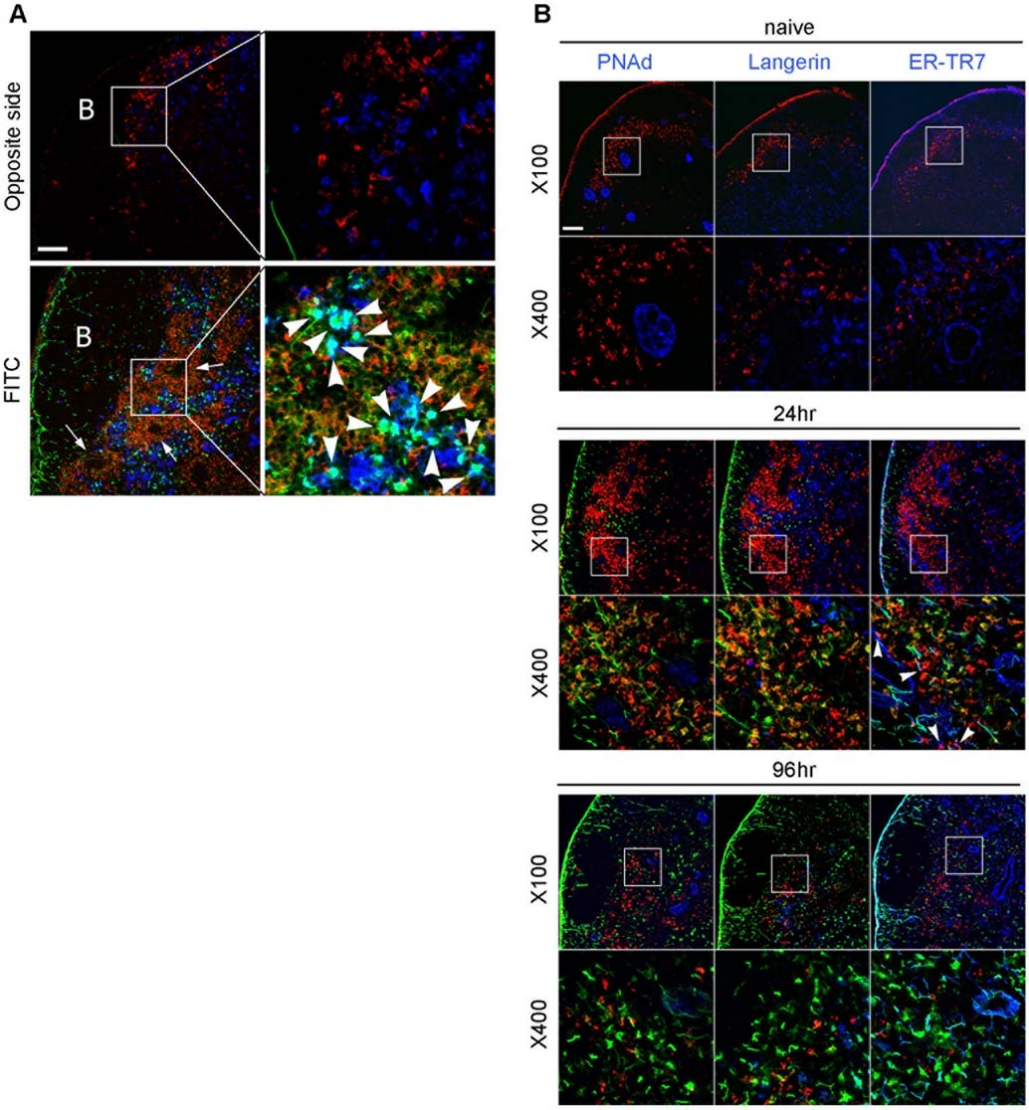
Immunohistochemical localization of MGL2^+^ DDCs, LCs and LN conduits. (A) Distribution of MGL2 (red) and Langerin (blue) in a draining (top) or a non-draining (bottom) LNs 24 h after FITC (green) sensitization. Arrows indicate venule-like structures in the CR. Arrowheads indicate the Langerin^+^ cells loaded with FITC. Almost all the MGL2^+^ DDCs were FITC^+^ and shown in yellow when merged. B: B cell follicles. Scale bar, 100 µm. (B) Distribution of MGL2 (red), PNAd (HEV), Langerin (LC) or ER-TR7 antigen (FRN) (blue in each panel) in the sensitization process. Areas indicated by the squares are magnified in the lower panels. FITC is shown in green. CR associated with HEV in a naive LN is adjacent to MGL2^+^ DDC only at its follicular side but not at the cortical side. Arrowheads indicate association of MGL2^+^ DDCs with ER-TR7^+^ FRN around HEV-associated CR. Scale bar, 100 µm.

It was previously unknown whether LCs and DDCs had similar spatial accessibility to naive T cells in the draining LNs. Because MGL2^+^ DDCs were found localized in the outer areas of the T cell cortex under steady state conditions (Figure 2A and B), the distribution of MGL2^+^ DDCs and Langerin^+^LCs within the LNs was examined during the FITC sensitization period. As expected from the MGL2 distribution shown in Figure 2, MGL2^+^ DDCs were observed mainly in the outer T cell cortex, the T- and B-cell boundaries, both in the draining and non-draining LN 24 h after sensitization (Figure 6A). Interestingly, the MGL2^+^ DDCs were completely segregated from the Langerin^+^ LCs, which were scattered through the deep T cell cortex, indicating that the distribution of these two DC subsets is distinct both under naive and sensitized conditions. We also noted the presence of MGL2^−^ Langerin^+^ DCs loaded with very bright FITC, though their number was relatively small (Figure 6A). They probably reflect the presence of FITC^+^ MGL2^−^ Langerin^+^ DCs detected by flow cytometry in the draining LNs 24 h after sensitization (Figure 5B). These cells might be Langerin^+^ LCs reside in the LNs at the time of sensitization and took up FITC in the LNs. They might also represent Langerin^+^ DDCs reported in Langerin-EGFP transgenic mice [19]–[21].

The segregation of MGL2^+^ DDCs and Langerin^+^ LCs appeared very similar to the distribution of the hapten-incorporated Langerin^−^ and Langerin^+^ DCs reported previously [12], further confirming each other that these cells were derived from the dermis. In the LNs 24 h after sensitization, almost all the MGL2^+^ DDCs were co-localized with FITC, indicating that MGL2^+^ DDCs incorporated FITC at this time while only a few LCs incorporated the hapten (Figure 6A). In addition, these FITC^+^ MGL2^+^ DDCs markedly accumulated around the venule-like structures, and Langerin^+^ LCs seemed to be relegated from these areas at 24 h after sensitization (Figure 6A). To identify the structures to which MGL2^+^ DDCs accumulated in detail, localizations of MGL2^+^ DDCs, high endothelial venules (HEV), LCs, and fibroreticular networks (FRN) in the LNs after sensitization were examined. At all time points examined, MGL2^+^ DDCs localized close to peripheral lymph node addressin (PNAd)^+^ HEV. Some of the HEV were associated with ER-TR7^+^ fibroreticular cells (Figure 6B) which were known to constitute an HEV-associated fibroreticular network termed as cortical ridge (CR) [41]. These results indicate that MGL2^+^ DDCs distribute around the CR regardless of the sensitization status, while Langerin^+^ LCs migrate further into the T cell cortex. Thus, MGL2 is likely to serve as a specific marker for a subset of DDCs and assists direct visualization of these cells *in situ*.

### MGL2^+^ DDCs are sufficient for induction of CHS

**Figure 7:**
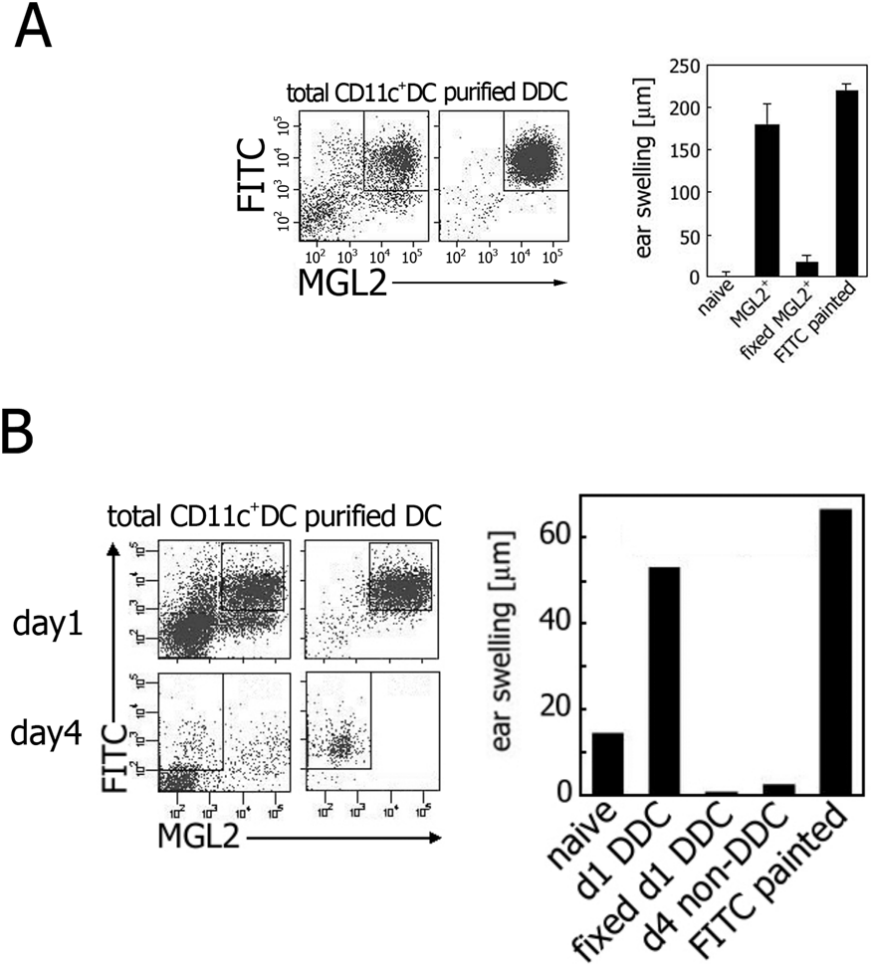
Induction of FITC-specific CHS in mice adoptively transferred with FITC^+^ MGL2^+^ DDC. (A) The left panels show the gating to sort FITC^+^ MGL2^+^ cells from the MACS-purified CD11c^+^ cells. The purity of FITC^+^ MGL2^+^ DDCs was approximately 98%. Ear swellings in mice transferred with FITC^+^ MGL2^+^ DDCs (“MGL2^+^”, *p*<0.005), transferred with glutaraldehyde-fixed FITC^+^MGL2^+^ DDC (“fixed MGL2^+^”, difference not statistically significant), sensitized directly with FITC painting (“FITC painted”, *p*<0.005), or untreated (“naive”) are shown in the graph. Each bar indicates mean±SD obtained from three independent recipients respectively. (B) The left panels show the gating strategy to sort FITC^+^MGL2^+^DDCs from the day 1 LNs or FITC^+^MGL2^−^DCs from the day 4 LNs. The purity of sorted cells was >90% when re-analyzed by FACS. Ear swelling in mice transferred with 5×10^4^ cells of FITC^+^MGL2^+^DDCs from the day 1 LNs (d1 DDC), glutaraldehyde-fixed FITC^+^MGL2^+^DDCs from the day 1 LNs (fixed d1 DDC) or FITC^+^MGL2^−^DCs from the day 4 LNs (d4 non-DDC) is shown in the right panel. The mice sensitized by FITC painting (FITC painted) and left untreated (naive) were used as positive and negative controls respectively. Each bar indicates the mean value from two recipients and the data are representative of two independent experiments.

Because MGL2^+^ DDCs apparently migrated and accumulated in the CR, where DC-T cell interaction and initial proliferation of antigen-specific T cells were known to take place [41], [42], we hypothesized that MGL2^+^ DDCs alone are likely to initiate CHS by presenting haptens to T cells. The availability of MGL2 as a marker for a DDC subset enabled us to directly address whether MGL2^+^ DDCs were sufficient in the induction of CHS by adoptive transfer experiments. FITC^+^ MGL2^+^ DDCs were isolated from the draining LNs of FITC-painted mice at day 1, and 7.5×10^4^ cells were transferred into the footpad of a naive recipient (Figure 6). Six days later, the recall responses in the recipients were elicited with FITC on the dorsal ear skin. Twenty-four hours after FITC painting, a significant ear swelling in the recipient of the MGL2^+^ DDCs was observed to a similar extent to the mice painted with FITC (Figure 7A). The swelling was virtually absent when glutaraldehyde-fixed FITC^+^ MGL2^+^ DDCs were transferred, suggesting that live cells were necessary to induce CHS in this system (Figure 7A). As an additional control, FITC^+^MGL2^−^DDCs isolated from the day 4 draining LNs were tested. This time point was chosen because LC migration was reported to peak around day 4 [12], [22], though we could not exactly address whether these transferred cells were LCs due to low Langerin expression on the cell surfaces preventing us from sorting experiments. When 5.0×10^4^ cells were transferred, FITC^+^MGL2^+^DDCs isolated from the day 4 draining LNs were able to induce CHS upon re-stimulation, whereas FITC^+^MGL2^−^DCs, putatively LCs, were not (Figure 7B). Although one could argue that the difference in CHS-inducing capacity between these two populations were due to difference in the efficiency of FITC incorporation and not to Ag-presentation and/or migration capability, the results shown in Figure 7 strongly suggest that MGL2^+^DDCs are potent inducers of CHS. It is possible that priming of T cells require participation of the third subset, e.g. CD8^+^DCs residing in LNs, as suggested by a previous report [22]. Thus, whether MGL2^+^DDCs alone are sufficient as APCs remained to be examined in future studies. From our finding that MGL2^+^ DDCs were earlier migrants in CHS sensitization and that they constituted the vast majority of the FITC-loaded cells in the LNs at the early sensitization phase (Figure 5), we concluded that expression of MGL2 is characteristic to a migratory DC subset sufficient for inducing CHS.

## Discussion

In the present study, selective expression of a C-type lectin, MGL2, in the majority of DDCs was demonstrated. Using this as a specific marker, the migration and the antigen presenting functions of MGL2^+^ DDCs in the initiation of CHS in the skin-draining LNs became evident. Previously, the surface phenotype differences between LCs and DDCs in cutaneous LNs were not clear because definitions for these cells were based on the anatomical comparison between cutaneous and mesenteric LNs [7], [10]. Only one molecule, Langerin, seemed to distinguish LCs from DDCs, though this marker was also expressed in CD8α^+^DCs [3], [43], [44]. However, specificity of this marker to distinguish LCs from DDCs in the skin-draining LNs was compromised by the discovery of Langerin^+^ DDCs [19]–[21].

In contrast to LCs, no marker has been identified for DDCs in mouse. Until Langerin^+^ DDCs were discovered, the DDCs in the cutaneous LNs were distinguished from the LCs by their relatively lower expression of CD205 and absence of Langerin [11], [12]. It was difficult to discriminate DDCs clearly from LCs, as both CD205^hi^ and CD205^int^ fractions sorted from the LN-derived DCs contained Langerin^+^ and Langerin^−^ DCs [12]. In addition, because of similarity between LCs and DDCs, differential expression of a particular marker between these two subsets was debatable. For instance, when Langerin^+^ LCs and Langerin^−^ DDCs in the skin-draining LNs were compared for the expression of CD11b, it was reported to be higher in Langerin^−^ DDCs [45], comparable in both DC subsets [11],or lower in DDCs [19]. Our results demonstrated that MGL2^+^DDCs in the skin-draining LNs expressed higher levels of CD8α and CD11b than Langerin^+^LCs (data not shown). Identification of MGL2 as a specific marker for a DDC subset enabled direct investigations of the phenotype and function of DDCs in the skin-to-LN immune system.

We concluded that MGL2 could be utilized as a marker for a DDC subset in skins and LNs based on the following observations. First, in the skin, MGL2^+^ cells represented the majority of dermal MHCII^hi^ CD11c^+^ cells. Second, MGL2^+^ cells in LNs showed a typical phenotype of interstitial DCs as reported previously [6], [7], [9], [10]. Third, MGL2^+^ DCs in the LNs were populations migrated from the skin and the migration was inhibited by PTX. The expression of MGL2 was stable upon the migration. In addition, judging from the immunohistochemistry and flow cytometric analyses of LNs, MGL2^+^ DDCs were a distinct population from Langerin^+^ LCs. The absence of Ly6G expression and the FITC-loading capacity after CHS sensitization suggested that MGL2^+^ DDCs were also a distinct population from inflammatory monocyte-derived DCs [37], [39], [40]. These results confirmed that MGL2 was exclusively expressed in the majority of the DDC subset (88±7% in dermal CD11c^+^MHCII^+^cells as shown in Figure 1C). We were not able to address whether MGL2^+^ DDCs were distinct from the Langerin^+^ DDCs in the physiological states, because the latter population was so far distinguishable from Langerin^+^ LCs only in bone marrow chimeric mice [19]–[21]. However, we assume that the majority of MGL2^+^ DDCs are distinct from Langerin^+^ DDCs, based on the nearly exclusive expression of MGL2 and Langerin in LNs by flow cytometry (Figure 3D) and immunohistochemistry (Figure 6). In organs other than the skin, MGL2^+^ cells are present in other stratified squamous epithelium of the mucosal lining of the vagina, esophagus and oral cavity but not in the spleen (data not shown), suggesting that MGL2 might be commonly expressed on a subset of tissue-resident and migrant DCs with a similar developmental background in each microenvironment. Although *Mgl2* mRNA transcription was reported to be upregulated in the alternative activation of MØs [46], MGL2^+^ DDCs seemed to be highly immunogenic *in vivo* as demonstrated in the present study.

MGL2^+^ DDCs were revealed to be early migrants from skins to LNs in the CHS sensitization process, which accounted for the appearance of hapten-loaded Langerin^−^ DCs in the draining LNs prior to LCs [12], [14], [22]. Thus, MGL2^+^ DDCs but not LCs were hypothesized to initiate adaptive immune responses to exogenous antigens in the skin. LCs were classically believed to act as the major APCs in CHS sensitization based on several findings [47] including incorporation of antigens [48], [49], correlation of CHS response with LC density at the sensitization site [50], presence of LCs with APC function in the LNs [15], and location of LCs at the surface of the body [51]. In these previous reports, however, identification of LCs was mainly based on the presence of the Birbeck granules characterized by electron microscopy, which was not applicable in studying cellular functions *in situ*. Some results presented in these previous reports might be explained if the population was represented by DDCs but not by LCs. Macatonia *et al.* stated in their report that FITC incorporation was the highest in LCs with Birbeck granules isolated from the LNs 24 h after FITC sensitization [15], whereas the period is when MGL2^+^ DDCs migrate into LNs in the present study. They also found that the capability to proliferate T cells in vitro was the highest with LCs isolated on day 1 but low with those isolated on day 4 [15]. However, our results, in conjunction with the results from the *in vivo* LC depletion studies showed that LCs were not required for the initiation of CHS [12], [17], [18]. These results suggest that MGL2^+^ DDCs but not LCs initiate adaptive immune responses to antigens invading the skin.

In addition to the kinetics of the migration of MGL2^+^ DDCs from the dermis to the LNs, the unique localization of MGL2^+^ DDCs within the LNs supports our hypothesis that MGL2^+^ DDCs are the predominant APCs in CHS. As reported previously [12], the FITC^+^MGL2^+^ DDCs were segregated from the LCs in the LNs 24 h after sensitization. Moreover, even in the steady state, MGL2^+^ DDCs were also physically and temporally separated from the LCs and accumulated in the outer T cell cortex, especially in the CR. This localization of MGL2^+^ DDCs was found to be highly unique among other DCs such as CD11c^+^ DC, CD205^+^ DCs and Langerin^+^ LCs, all of which were distributed throughout the T cell cortex [1], [52], [53]. Two possibilities could explain the distinct migration and distribution patterns of these DCs in the LNs. First, LCs migrate directly into the deep T cell cortex through different lymphatics from the ones that MGL2^+^ DDCs migrate through. The other possibility is that these two migrants share the same lymphatic routes to the LNs but are differently distributed within the LNs. The former possibility is not likely because many HEVs were surrounded by MGL2^+^ DDCs at their follicular sides and by the LCs at their cortical sides in naive LNs. It was likely that these DCs once migrated to the same areas in the CR, and only LCs migrated further into the deep T cell cortex while MGL2^+^ DDCs remained in the CR. The common route to the CR might be under the regulation of the chemokine receptor CCR7 because both CD11c^int^ CD40^hi^ DCs (population II in Figure 4A) and CD11c^hi^ CD40^hi^ DCs (population III: MGL2^+^ DDCs) seemed to be reduced in the CCR7-deficient mice [35] and also because CCR7 ligands might be concentrated on the CR stroma [41]. The preferential localization of MGL2^+^ DDCs in the CR is consistent with the idea that MGL2^+^ DDCs are the population responsible for initiation of the CHS response because the CR is the sites of interaction between DC and newly migrated T cells and the sites of the primary T cell proliferation *in vivo* [41], [42], [54], [55].

In conclusion, we have demonstrated that expression of MGL2 characterizes a DDC population sufficient for CHS initiation. Functional difference between MGL2^+^ DDCs and Langerin^+^ DCs in CHS and the molecular function of MGL2 in these cells should further be characterized.
